# Prevalence and Prognostic Value of Early Repolarization in Low Risk Surgical Patients

**DOI:** 10.1155/2015/309260

**Published:** 2015-07-13

**Authors:** Chiho Ota, Sin-nosuke Shiono, Yuji Fujino, Takahiko Kamibayashi, Yukio Hayashi

**Affiliations:** ^1^Department of Anesthesiology, Osaka University Faculty of Medicine, Suita, Osaka 565-0871, Japan; ^2^Anesthesiology Service, Sakurabashi-Watanabe Hospital, 2 Chome-4-32 Umeda, Kita-ku, Osaka 530-0001, Japan

## Abstract

Recent epidemiological studies documented that early repolarization may be associated with increased risk of serious cardiac events, including cardiac death. Little is known about the prognostic significance of this pattern in low risk surgical patients. This retrospective study included 3028 patients over 18 years of age and with ASA class I and II risk, undergoing noncardiac elective surgery. We followed the patients for one year. Early repolarization in preoperative ECG was found in 219 patients (7.2%) and patients with early repolarization were more likely to be male and younger. Newly observed cardiac events were significantly higher in the early repolarization group (1.37% versus 0.21%; *P* = 0.003). Multivariate regression analysis reveals that early repolarization (odds ratio: 6.019, *P* = 0.013) significantly increased newly observed cardiac events. Our retrospective study suggests that low risk surgical patients with early repolarization have statistically higher opportunity of newly observed cardiac events within one year after surgery.

## 1. Introduction

**Figure 1:**
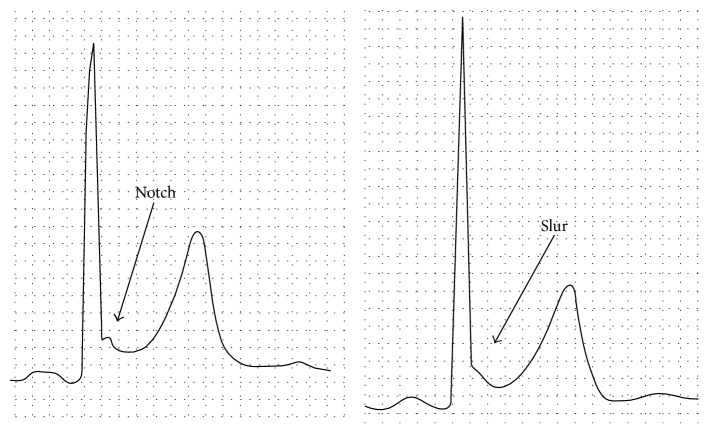
Typical examples of early repolarization.

Early repolarization is characterized by J point elevation with QRS notching or slurring (Figure 1) in inferior and/or lateral leads and it has been generally considered to be benign [1, 2]. However, recent epidemiologic studies documented that this electrocardiographic pattern may be associated with increased risk of cardiac events, including cardiac death [3–6]. This ECG pattern is reported to be 1% to 6% of all population [1, 3, 5]. So it may not be rare to have a chance to anaesthetize a patient with this ECG pattern. So far, little has been reported about the prognostic significance of this ECG pattern in low risk surgical patients. This retrospective study was designed to assess the prevalence of the early repolarization pattern and its long-term prognosis in low risk surgical patients.

## 2. Materials and Methods 

This retrospective study was approved by our institutional ethics committee (#08307) and written informed consent from each patient was omitted because of the retrospective study design.

This study included 3304 adult patients over 18 years of age with ASA class I or II class who underwent general anesthetic management for noncardiac elective operations in our hospital from 1 January 2010 to 31 December 2010 and whose clinical and outcome data were able to be ascertained through hospital electrical records and provincial records until more than one year after surgery. Although eligible patients in this study were low ASA class (I or II), we added further following exclusion criteria before anesthesia: structural or coronary heart disease, atrial and ventricular arrhythmias, left and right bundle branch block, chronic obstructed pulmonary disease, and hepatic (liver enzymes more than 100 units) and renal dysfunction (induction of hemodialysis).

After arrival of the patient in the operating room, monitoring, including electrocardiography, noninvasive arterial pressure monitoring, pulse oximetry, and capnography, was established. Then, after preoxygenation, general anaesthesia was induced with intravenous thiamylal or propofol. Patients were paralyzed with vecuronium or rocuronium and were intubated by a resident anesthesiologist managing the patient. Anesthesia was maintained with propofol or sevoflurane with combination of opiates (fentanyl and remifentanil).

We examined all preoperative ECG records. Using criteria similar to those of Haïssaguerre et al. [5], we defined early repolarization as at least 0.1 mV elevation of the J point or ST segment, with notching or slurring in at least 2 inferior (II, III, and aVF) and/or lateral leads (I, aVL, and V4–6) (Figure 1). All ECG records were checked by one anesthesiologist. The anterior precordial leads (V1 to V3) were excluded from analysis to avoid the inclusion of patients with right ventricular dysplasia or Brugada syndrome. The subjects were followed up for at least one year after surgery. Clinical and outcome data were obtained through hospital electrical records and provincial records until more than one year after surgery and the presence of the following end points was ascertained. The primary end point is death from any cause and from cardiac causes within one year after anesthesia. The second end point is newly observed cardiac events found within one year after anesthesia, including arrhythmias, angina pectoris, myocardial infarction, and congestive heart failure, but temporary arrhythmias and myocardial ischemic changes after anesthesia are not included. The following variables were obtained from the medical record: demographic characteristics (age, sex, height, and weight), preoperative medical history such as hypertension and diabetes mellitus, preoperative smoking status, preoperative cardiac medication including *α* and *β* blockers, Ca channel blockers, angiotensin converting enzyme inhibitors, angiotensin receptor blockers and nitrates, preoperative early repolarization, risk of surgery, and duration of anesthesia. The smoking status was categorized as current, ex-smoker, and never-smoker. A current smoker was defined as one who had smoked within 2 weeks prior to the operation, an ex-smoker was defined as one whose duration of smoke-free period was >2 weeks prior to the operation, and a never-smoker was defined as one who had never smoked. The risk of surgery was stratified as low, intermediate, and high according to ACC/AHA 2007 guidelines on perioperative cardiovascular evaluation and care for noncardiac surgery; that is, low risk surgeries include endoscopic procedures, superficial procedures, cataract surgery, beast surgery, and ambulatory surgery; intermediate risk surgeries include intraperitoneal and intrathoracic surgery, carotid endarterectomy, head and neck surgery, orthopedic surgery, and prostate surgery; and high risk surgeries include vascular surgeries [7].

Based on 1019 cases including 113 cases of early repolarization from January 2010 to April 2010, sample size was calculated to be 3080 for detecting more newly observed cardiac events one year after surgery in patients with preoperative early repolarization at the significant level of 0.05 with a statistical power of 80%. Thus, we included cases of one year to clear the sample size.

Continuous variables are presented as mean ± SD and categorical variables are presented as percentage. After univariate analyses, logistic regression analysis was performed to assess the one-year prognosis of covariates. Univariate predictor variables with *P* values less than 0.1 were considered for inclusion in multivariate models. Unpaired *t*-test and *χ* ^2^ analyses were performed to compare between two groups in the presence and absence of preoperative early repolarization as appropriate. All analyses were conducted with SPSS version 14.0. *P* < 0.05 was considered statistically significant.

## 3. Results and Discussion

**Figure 2:**
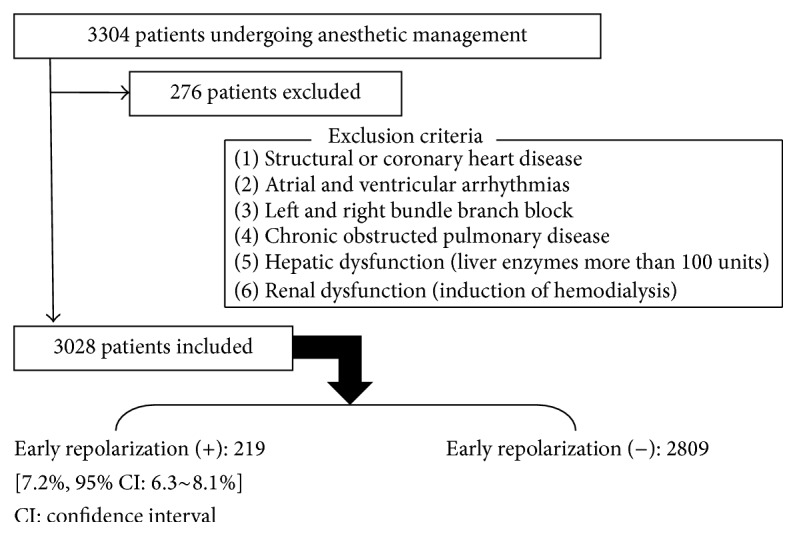
Patient inclusions and exclusions.

This study enrolled 3304 patients, but 276 patients were applicable to the exclusion criteria (Figure 2). Therefore, 3028 patients were included and 219 patients had early repolarization in preoperative ECG. Then, the incidence of this ECG pattern was 7.2% (95% confidence interval: 6.3~8.1%) (Figure 2). The average duration of follow-up was 12.03 ± 1.34 months (mean ± SD). The characteristics and outcome of the subjects are summarized in Tables 1 [Table tab2]–3. Subjects with early repolarization were more likely to be male and younger than those without early repolarization. In addition, hypertension was significantly more frequent in those without early repolarization and preoperative cardiac mediation was also more frequent in this population. Although the death from any cause or cardiac causes was not significantly different between the groups, newly observed cardiac events were significantly higher in the early repolarization group. Univariate analysis showed that age (*P* = 0.018), male (*P* = 0.06), and early repolarization (*P* = 0.009) were significantly associated with newly observed cardiac events within one year after surgery (Table 4). The logistic regression analysis shows that age and early repolarization were significant predictors but male was not (Table 5).

The principal finding of this study is that the presence of early repolarization before anesthesia is a marker of increased risk of newly observed cardiac events one year after surgery in low risk surgical patients.

The presence of J point elevation on standard 12-lead electrocardiography has generally been considered as an innocuous finding in healthy persons [8]. Recently, however, some clinical studies documented that this ECG pattern may be associated with an increasing risk of serious arrhythmias and death from cardiac causes [3–6, 9], although the clinical significance of this ECG is still controversial [1.8.10]. So far, the prognostic value of this ECG pattern in low risk surgical patients has not been reported. In this study, our multivariate statistical analysis demonstrated that early repolarization is associated with newly developed cardiac events one year after surgery (Tables 4 and 5).

The present data showed that percentage of cardiac death and newly observed cardiac events in the early repolarization group remained at 0.46 and 1.37%, respectively. These percentages are considerably lower than previous epidemiological studies [3, 4, 6, 9, 10]. We followed the patients for only one year. So we may expect the incidence to be similar to the previous epidemiological studies by a follow-up period of much longer duration.

With regard to the clinical significance of early repolarization in anesthetic management, present knowledge may be inadequate for preoperative risk stratification and detailed counseling of asymptomatic individuals with early repolarization patterns. We may show the statistical association of early repolarization with the newly observed cardiac events, but the number of events is small (three cases in the early repolarization group). Thus, we would be prudent to mention clinical causality between early repolarization and the cardiac events. Nevertheless, our study is the first clinical trial documenting a significant relationship between preoperative early repolarization and cardiac events after surgery. Accumulation of further clinical data might establish clinical significance of early repolarization as a preoperative risk factor.

According to previous studies, prevalence of early repolarization has been reported to be 1% to 6% [1–3, 5]. Our data show that early repolarization occurs in 7.2% of patients undergoing noncardiac surgery. Thus, this is slightly more than the data previously reported, although one recent report by Sinner et al. [6] documented that the overall prevalence of early repolarization was 13.1%. One possible reason may be the fact that the subjects are Japanese populations. One clinical report form Japan [10] has documented that the incidence of early repolarization is higher than that in European and American people. On the other hand, in agreement with the data from previous clinical reports [1, 11, 12], more males and younger patients were found in our early repolarization group (Table 1).

In general, early repolarization may mean the presence of a form of transmural electrical heterogeneity of ventricular repolarization. So, recent clinical reports have focused on a vulnerability to arrhythmias in patients with early repolarization. However, we have extended the end point to newly observed cardiac events without limiting it to arrhythmias. In our study population, we included no subjects with structural heart disease, but the results may document that early repolarization was a marker of an increased risk of cardiac events after anesthesia among low risk surgical patients. Presumably, early repolarization might reflect the presence of some potentially existing cardiac injury which is not presently evident or realized preoperatively but appears after potential stress associated with surgical invasion and/or anesthesia.

It is well known that aging facilitates organ dysfunction, resulting in an increase in perioperative and postoperative mortality and major morbidity [13]. Our data also showed that age was a significant risk factor for newly observed cardiac events one year after anesthesia like early repolarization. Interestingly, younger patients were found in our early repolarization group. Thus, a hazard effect of early repolarization is seen despite it being more common in younger subjects.

Hypertension, diabetes mellitus, and smoker are also known to be associated with an increased incidence of heart diseases [13]. However, the present data did not show significant effect of these variables, although the tendency was seen (Table 4). Since observation period of our study was just one year and the number of cardiac events (3 versus 6) was small, longer term observation and increased number of patients may be required to elucidate the clinical significance of these variables.

We have to discuss potential limitations in our study. First, although our results may reach the statistical significance about the newly observed cardiac events (Table 5), the numbers of events in each group were small (3 versus 6). Thus, the clinical significance of our results would be interpreted with caution. Second, we did not assess other cardiovascular risk factors, such as hyperlipidemia and left ventricular hypertrophy in this study, because plasma cholesterol and cardiac echography are not included in our routine preoperative laboratory examination for low risk patients. Third, although we found 9 cases of newly observed cardiac events in this study, we could not completely deny the preoperative presence of structural heart diseases in these subjects because we did not perform preoperative echocardiography in our subjects because of the retrospective design. Fourth, previous clinical studies have documented that J wave of lateral leads has higher diagnostic value than V4~6 leads [4, 9]. Actually, we found only three cases of newly observed cardiac events and one cardiac death in the early repolarization group. Thus, our subjects are not enough to perform further risk stratification in our study design.

## 4. Conclusions

Our retrospective study suggests that low risk surgical patients with early repolarization have statistically higher opportunity of newly observed cardiac events within one year after surgery.

## Figures and Tables

**Table 1:** Characteristics of the subjects with and without early repolarization pattern in preoperative ECG.

	Early repolarization (+)	Early repolarization (−)	*P* value
Number of subjects	219	2809	
Age (yr)	53.6 ± 18.1^*∗*^	56.6 ± 16.0	0.008
Male	154 (70.3%)^*∗*^	1170 (41.7%)	<0.001
Duration of anesthesia (min)	268 ± 190	267 ± 183	0.95
	Low: 82 (37.4%)	Low: 916 (32.6%)	
Surgical risk	Intermediate: 136 (62.1%)	Intermediate: 1887 (67.2%)	0.25
	High: 1 (0.5%)	High: 6 (0.2%)	
Hypertension	37 (16.9%)^*∗*^	666 (23.7%)	0.021
Diabetes mellitus	18 (8.2%)	260 (9.3%)	0.27
Current smoker	29 (13.2%)	321 (11.4%)	0.43
Ex-smoker	23 (10.5%)	233 (8.3%)	0.27
Medication	33 (15.1%)^*∗*^	599 (21.3%)	0.023

Age is expressed as mean ± SD. Other values are expressed as number (%).

^*∗*^ *P* < 0.05, compared with the no early repolarization group.

**Table 2 tab2:** Outcome within one year after the operation of the subjects with and without early repolarization pattern in preoperative ECG.

	Early repolarization (+)	Early repolarization (−)	*P* value
Number of subjects	219	2809	
Death from any cause	8 (3.65%)	53 (1.89%)	0.07
Death from cardiac causes	1 (0.46%)	2 (0.07%)	0.08
Newly observed cardiac events	3 (1.37%)^*∗*^	6 (0.21%)	0.003

The values are expressed as number (%).

^*∗*^ *P* < 0.05, compared with the no early repolarization group.

**Table 3:** The summary of subject with death from any cause and newly observed cardiac events.

	Early repolarization (+)	Early repolarization (−)
Cause of death	**8**	**53**
Primary disease	7	41
Myocardial infarction	1	2
Sepsis	0	1
Pneumonia	0	2
Subarachnoid hemorrhage	0	1
Carcinoma (unknown at the operation)	0	1
Traffic accident	0	1
Unknown	0	4
Newly observed cardiac events	**3**	**6**
Angina	0	1
Myocardial infarction	1	3
Newly developed arrhythmias	2	2

**Table 4:** Univariate analyses of potential predictors of increased newly observed cardiac events.

Variable	Odds ratio	95% confidence interval	*P* value
Age	1.074	1.012–1.139	0.018
Male	4.523	0.938–21.809	0.06
Height	1.043	0.961–1.131	0.312
Weight	1.004	0.994–1.014	0.45
Early repolarization	6.488	1.612–26.123	0.009
Surgical risk	0.965	0.244–3.814	0.959
Duration of anesthesia	1.001	0.998–1.004	0.517
Hypertension	2.655	0.711–9.915	0.146
Diabetes mellitus	2.840	0.587–13.735	0.194
Current smoker	2.186	0.452–10.564	0.331
Ex-smoker	3.110	0.643–15.050	0.158
Cardiac medication	1.900	0.474–7.618	0.365

**Table 5:** Multivariate association with increased newly observed cardiac events: logistic regression analysis.

Variable	Odds ratio	95% confidence interval	*P* value
Age	1.076	1.011–1.145	0.021
Male	3.261	0.662–16.056	0.146
Early repolarization	6.019	1.451–24.969	0.013
